# Arachidonic Acid–Induced Dilation in Human Coronary Arterioles: Convergence of Signaling Mechanisms on Endothelial TRPV4‐Mediated Ca^2+^ Entry

**DOI:** 10.1161/JAHA.113.000080

**Published:** 2013-06-21

**Authors:** Xiaodong Zheng, Natalya S. Zinkevich, Debebe Gebremedhin, Kathryn M. Gauthier, Yoshinori Nishijima, Juan Fang, David A. Wilcox, William B. Campbell, David D. Gutterman, David X. Zhang

**Affiliations:** 1 Department of Medicine, Medical College of Wisconsin, Milwaukee, WI (X.Z., N.S.Z., Y.N., D.D.G., D.X.Z.); 2 Cardiovascular Center, Medical College of Wisconsin, Milwaukee, WI (X.Z., N.S.Z., D.G., Y.N., D.D.G., D.X.Z.); 3 Department of Physiology, Medical College of Wisconsin, Milwaukee, WI (D.G.); 4 Department of Pharmacology and Toxicology, Medical College of Wisconsin, Milwaukee, WI (K.M.G., W.B.C.); 5 Department of Pediatrics, Medical College of Wisconsin, Milwaukee, WI (J.F., D.A.W.); 6 Children's Research Institute, Children's Hospital of Wisconsin, Milwaukee, WI (J.F., D.A.W.); 7 Zablocki Veterans Affairs Medical Center, Milwaukee, WI (D.D.G.)

**Keywords:** arachidonic acid, calcium, endothelium‐dependent hyperpolarization, endothelium‐derived hyperpolarizing factors, membrane potential, transient receptor potential V4

## Abstract

**Background:**

Arachidonic acid (AA) and/or its enzymatic metabolites are important lipid mediators contributing to endothelium‐derived hyperpolarizing factor (EDHF)–mediated dilation in multiple vascular beds, including human coronary arterioles (HCAs). However, the mechanisms of action of these lipid mediators in endothelial cells (ECs) remain incompletely defined. In this study, we investigated the role of the transient receptor potential vanilloid 4 (TRPV4) channel in AA‐induced endothelial Ca^2+^ response and dilation of HCAs.

**Methods and Results:**

AA induced concentration‐dependent dilation in isolated HCAs. The dilation was largely abolished by the TRPV4 antagonist RN‐1734 and by inhibition of endothelial Ca^2+^‐activated K^+^ channels. In native and TRPV4‐overexpressing human coronary artery ECs (HCAECs), AA increased intracellular Ca^2+^ concentration ([Ca^2+^]_i_), which was mediated by TRPV4‐dependent Ca^2+^ entry. The AA‐induced [Ca^2+^]_i_ increase was inhibited by cytochrome P450 (CYP) inhibitors. Surprisingly, the CYP metabolites of AA, epoxyeicosatrienoic acids (EETs), were much less potent activators of TRPV4, and CYP inhibitors did not affect EET production in HCAECs. Apart from its effect on [Ca^2+^]_i_, AA induced endothelial hyperpolarization, and this effect was required for Ca^2+^ entry through TRPV4. AA‐induced and TRPV4‐mediated Ca^2+^ entry was also inhibited by the protein kinase A inhibitor PKI. TRPV4 exhibited a basal level of phosphorylation, which was inhibited by PKI. Patch‐clamp studies indicated that AA activated TRPV4 single‐channel currents in cell‐attached and inside‐out patches of HCAECs.

**Conclusions:**

AA dilates HCAs through a novel mechanism involving endothelial TRPV4 channel‐dependent Ca^2+^ entry that requires endothelial hyperpolarization, PKA‐mediated basal phosphorylation of TRPV4, and direct activation of TRPV4 channels by AA.

## Introduction

Endothelial cells (ECs) play a crucial role in the regulation of vascular tone through the release of a number of vasoactive factors, particularly relaxing factors such as nitric oxide (NO), prostaglandin I_2_ (PGI_2_), and endothelium‐derived hyperpolarizing factor (EDHF).^1^ Tonic release of these relaxing factors counteracts resting smooth muscle tone, whereas stimulated production of relaxing factors in response to chemical agonists (eg, bradykinin and acetylcholine) and mechanical stimuli (eg, shear stress) induces vasodilation. Although the importance of a specific endothelial relaxing factor may vary in different species and vascular beds, EDHF‐ or endothelium‐dependent hyperpolarization (EDH)–related dilation in general is more predominant in small resistance arteries and in disease states in which the contribution of other factors such as NO diminishes. With regard to the chemical identity of EDHFs, there is evidence that epoxyeicosatrienoic acids (EETs), cytochrome P450 (CYP) epoxygenase‐derived metabolites of arachidonic acid (AA), and hydrogen peroxide (H_2_O_2_), a reactive oxygen species (ROS), serve as 2 prominent EDHFs in a variety of vascular beds from both experimental animals and humans.^2–3^ Using human coronary arterioles (HCAs) from subjects with coronary artery disease (CAD), we have recently demonstrated that H_2_O_2_ represents a key EDHF responsible for flow‐mediated dilation and contributing to bradykinin‐induced dilation.^4–7^ Intriguingly, AA‐derived EETs also seem to be involved in EDH‐related dilation since blockade of CYP pathway also markedly reduces the dilation of HCAs to flow as well as to exogenous AA.^8–9^ Given that CYP activity is directly inhibited by H_2_O_2_,^10^ it remains largely unsolved how both H_2_O_2_ and AA‐derived EETs relate to EDH‐dependent dilation in HCAs.

An increase of intracellular calcium concentration ([Ca^2+^]_i_) is a critical signaling event in the release of endothelial relaxing factors.^1–2^ Recent studies from several laboratories including ours have implicated a Ca^2+^‐permeable cation channel from the transient receptor potential (TRP) channel family, TRP vanilloid type 4 (TRPV4), as having an important role in flow‐ and agonist‐induced dilation in several rodent vascular beds.^11–17^ Data from our laboratory also indicate that TRPV4‐mediated Ca^2+^ entry is involved in the release of ROS in coronary ECs and in flow‐induced dilation in HCAs.^18^ Despite extensive evidence supporting the role of TRPV4 in endothelium‐dependent vasodilation, the mechanisms by which extracellular stimuli activate endothelial TRPV4 channels and cause subsequent vasodilation remain incompletely understood. Several previous studies have indicated that CYP epoxygenase metabolites may act as endogenous activators of TRPV4 and thus contribute to AA‐induced Ca^2+^ entry in mouse and rat vascular cells.^19–22^ However, whether endothelial TRPV4 mediates the functional vasodilator effect of AA has not been directly examined. Furthermore, the role of these metabolites in TRPV4 activation in human vessels is not known.

The objectives of the present study were to determine the role of the endothelial TRPV4 channel in AA‐induced dilation of HCAs and in particular to elucidate the underlying mechanism by which the TRPV4 channel is activated by AA and/or its metabolites in human coronary ECs. Our results provide the first evidence that, in HCAs, AA‐induced dilation depends on endothelial TRPV4 channel‐mediated Ca^2+^ entry. We also demonstrate that this Ca^2+^ entry through TRPV4 channels in coronary ECs is mediated by a previously unidentified mechanism requiring coordination of endothelial hyperpolarization, protein kinase A (PKA)–mediated basal phosphorylation of TRPV4 and direct activation of TRPV4 channels by AA.

## Methods

### Tissue Acquisition

Fresh human right atrial appendage specimens were obtained as discarded surgical specimens from patients undergoing cardiopulmonary bypass surgery.^4^ After surgical removal, atrial tissues were immersed in a cardioplegia solution and immediately transported to the laboratory. All protocols were approved by the local institutional review boards on the use of human subjects in research. Patient demographic data are summarized in Table S1. A total of 20 atrial appendages (from 20 patients) were used in this study.

### Cell Culture

Human coronary artery endothelial cells (HCAECs) were obtained from Lonza (Walkersville, MD) and maintained in a full‐growth medium (EGM‐2MV from Lonza) according to the manufacturer's protocols. Cells were cultured in a humidified 37°C, 5% CO_2_ incubator, split at 90% to 95% confluence, and used between passages 5 and 7.

### Vascular Reactivity

HCAs (200 to 300 μm) were dissected from the atrial tissue and mounted in a wire myograph (Danish Myo Technology A/S, Denmark) as previously described.^6,23^ In some experiments, HCAs (100 to 200 μm) were cannulated with 2 glass micropipettes, and the internal diameter of arterioles was measured with a video system.^6,15^ Arterioles were precontracted with endothelin‐1, and relaxation responses to cumulative concentration of arachidonic acid (10^−9^ to 10^−6^ mol/L) were determined in the presence or absence of RN‐1734 (20 μmol/L), a specific antagonist of TRPV4. To examine the role of smooth muscle hyperpolarization in AA‐induced dilation, arterioles were preconstricted with high‐K (80 mmol/L K^+^) Krebs. To further determine the K^+^ channels involved in AA‐induced dilation, responses to AA were examined before and after 30‐minute incubation with TRAM‐34 (1 μmol/L), an inhibitor of the intermediate‐conductance Ca^2+^‐activated K^+^ channel (IK_Ca_), and apamin (1 μmol/L), an inhibitor of the small‐conductance K_Ca_ channel (SK_Ca_).

At the end of each experiment, papaverine (10^–4^ mol/L), an endothelium‐independent vasodilator, was added to determine the maximal dilation for normalization of dilator responses. Vasodilator responses are expressed as percentage of maximal relaxation relative to endothelin‐1 constriction, with 100% representing full relaxation to basal tension or the maximal diameter.

### Lentiviral Transgene Expression

The human TRPV4 (NM_021625) full‐length cDNA clone was obtained from Origene and shuttled into a pCMV6 mammalian expression vector, resulting in a TRPV4 fusion protein with its COOH‐terminus tagged with turbo green fluorescent protein (GFP).^15^ The turbo GFP has an excitation maximum of 482 nm, and therefore its fluorescence can be simultaneously measured in cells with that of the UV‐excitable dye fura‐2 (used in calcium imaging as below). The TRPV4‐GFP construct was excised from pCMV6, and subcloned into an HIV type‐1 vector, pWPTS‐GFP (gift from D. Trono, University of Geneva, Switzerland), replacing the GFP cDNA cassette. The nucleotide sequence of the TRPV4‐GFP construct was verified by direct DNA sequencing. Recombinant lentiviruses were produced from HKE‐293T cells cotransfected with 3 plasmids, pWPTS‐TRPV4‐GFP, pCMVΔR8.91, and pCI‐VSV‐G, using the calcium phosphate precipitation method as previously described.^24^ Lentiviral supernatants were concentrated 500‐fold by low‐speed centrifugation (7000*g* overnight) and stored frozen at −80°C until use. Virus titer was determined by real‐time quantitative reverse‐transcriptase PCR measuring copies of proviral DNA integrated into the genome of circulating murine mononuclear cells. Absence of replication‐competent virus particles in lentiviral stocks was confirmed by an extended marker rescue assay.

For TRPV4 overexpression experiments, HCAECs at passage 6 were grown to 50% to 60% confluence before being transduced with recombinant lentiviruses. Cells were split at a ratio of 1:4 to 1:8 into glass‐bottom dishes or coverslips 24 hours after transduction or when cells reached 80% to 90% confluence. To minimize potential cellular calcium overload from TRPV4 overexpression, the concentration of calcium in the culture medium was reduced to 0.4 to 0.6 mmol/L 48 hours after transduction by the addition of EDTA (1.2 mmol/L), and the medium pH was readjusted. Cells were used for calcium imaging 3 to 4 days after transduction and for patch‐clamp experiments 4 to 6 days after transduction.

### Calcium Imaging

HCAECs were plated onto 35‐mm glass‐bottom petri dishes and grown to 60% to 70% confluence. Cells were loaded with fura‐2 AM (5 μmol/L; Molecular Probes) at room temperature for 30 minutes in a modified Hanks balanced salt solution (HBSS) that contained (in mmol/L): 123 NaCl, 5.4 KCl, 1.6 CaCl_2_, 0.5 MgCl_2_, 0.4 MgSO_4_, 4.2 NaHCO_3_, 0.3 NaH_2_PO_4_, 0.4 KH_2_PO_4_, 5.5 glucose, and 20 HEPES (pH 7.4 with NaOH). Nominal Ca^2+^‐free HBSS was prepared by adding 1 mmol/L EGTA into HBSS without Ca^2+^, and the pH was adjusted to 7.4 with NaOH. A fura‐2 assay was used to monitor cytosolic Ca^2+^ signals as previously described.^15^ Fluorescence images were acquired for 20 to 30 minutes every 3 seconds in cells treated with 4α‐PDD (a specific TRPV4 agonist; 1 to 5 μmol/L), GSK1016790A (a specific TRPV4 agonist; 10 nmol/L), AA (3 μmol/L), palmitate (0.3 to 3 μmol/L), arachidic acid (0.1 to 10 μmol/L), EETs (3 to 10 μmol/L), valinomycin (a K^+^‐selective ionophore; 2 or 5 μmol/L), or forskolin (a PKA activator; 10 μmol/L). In some experiments, cells were pretreated for 20 to 30 minutes with the following chemicals at the indicated concentrations: TRPV4 inhibitors—RN‐1734 (20 μmol/L), HC‐067047 (1 μmol/L), ruthenium red (1 μmol/L); CYP450 inhibitors—17‐ODYA (10 μmol/L), ETYA (30 μmol/L), MS‐PPOH (30 μmol/L); EET antagonist 14,15‐EEZE (10 μmol/L); protein kinase C (PKC) inhibitor GF 109203X (1 μmol/L); PKA inhibitor PKI (1 μmol/L). Experiments were performed at 37°C for native HCAECs, and at room temperature (22°C to 25°C) for HCAECs overexpressing hTRPV4. [Ca^2+^]_i_ was calculated according to the following equation: [Ca^2+^]_i_=K_d_ (S_f,2_/S_b,2_) (R−R_min_)/(R_max_−R),^25^ where R is the ratio of fluorescence value at 340 nm (F_340_) over that at 380 nm (F_380_); R_min_ and R_max_ are minimal and maximal F_340_/F_380_ ratios, respectively; and S_f,2_/S_b,2_ represents the maximal and minimal signal intensity at 380 nm, respectively. K_d_ is the apparent dissociation constant of fura‐2 (224 nmol/L).

### Measurement of Plasma Membrane Potential (Em)

Fluorescent measurement of Em was performed using plasma membrane potential indicator (PMPI), a proprietary component of the FLIPR Membrane Potential Assay kit (Molecular Devices, R‐8128). An individual vial of the blue indicator dye was reconstituted in 1 mL of distilled water (PMPI stock), dispensed into 50‐μL aliquots, and stored at −20°C. For Em assays, cells were loaded with PMPI (5 μL of stock diluted in 1 mL of HBSS) for 30 minutes at 37°C and then transferred to the stage of an inverted microscope for fluorescence measurements. PMPI fluorescence was generated using an excitation wavelength of 480 nm and an emission wave wavelength of 550/40 nm. Images were acquired every 5 seconds under ×200 magnification. Background fluorescence was subtracted from each image. Cells were treated with vehicle, AA (3 μmol/L), or 4α‐PDD (3 μmol/L). Valinomycin (2 μmol/L) was used as a positive control to indicate Em hyperpolarization.

### Immunoprecipitation and Immunoblotting

Endothelial cells expressing the TRPV4‐GFP transgene were treated as specified in figure legends, rinsed with PBS, and solubilized in a lysis buffer (50 mmol/L Tris‐HCl, 150 mmol/L NaCl, 1.0% Nonidet P‐40, 0.5% sodium deoxycholate, 1 mmol/L EDTA [pH 7.5]) supplemented with protease and phosphatase inhibitors, followed by immunoprecipitation using a Protein A/G Agarose Immunoprecipitation kit (Roche Applied Science). In brief, cell lysates were centrifuged at 12 000*g* for 10 minutes, and protein samples (250 μg) were precleared with 25 μL of protein A‐agarose beads. The cleared supernatants were mixed with 1 μg of a rabbit polyclonal anti‐GPP antibody (Evrogen, AB513) for 1 hour and then with 25 μL of protein A‐agarose beads for an additional 3 hours to precipitate TRPV4‐GFP immunocomplexes. The immunoprecipitated proteins were collected by centrifugation at 12 000*g* for 20 seconds, washed 5 times, and eluted with 75 μL of Laemmli sample buffer (Bio‐Rad).

Protein samples (15 μL) were separated by SDS‐PAGE on 10% gels and transferred to PVDF membranes. Membranes were blocked by 10% BSA overnight at 4°C, and incubated with a rabbit monoclonal anti‐pS824 antibody (1:1000 in TBST with 5% BSA; #10001, Cell Signaling Technology) for 2 hours. This anti‐phospho‐serine/threonine antibody, which is raised against the motif RXRXXS*/T*, was used to identify phosphorylated serine‐824 of TRPV4 as previously reported.^26^ Blots were then washed with TBST prior to the addition of a goat anti‐rabbit antibody (1:5000 in TBST with 2% BSA; SC‐2054, Santa Cruz, CA) for 1 hour at room temperature. Membranes were developed using the ECL Plus reagent (Amersham). The same membrane was stripped in a stripping buffer solution (Pierce) and reprobed with a rabbit anti‐GFP antibody (1:15 000 in TBST with 2% milk; AB513, Evrogen) and then with a goat anti‐rabbit antibody (1:10 000 dilution in BS with 2% milk) to obtain the total TRPV4 signal.

### Metabolism of [^14^C]AA

HCAECs were plated onto 10‐cm petri dishes and grown to 70% to 80% confluence. Cells were incubated for 10 minutes at 37°C in HEPES buffer containing 0.1 μmol/L cold AA and 25 nmol/L (0.1 μCi) [^14^C]AA (Perkin Elmer Life Sciences, Boston, MA). A‐23187 (10 μmol/L, Sigma) was then added for an additional 15 minutes. In some experiments, 17‐ODYA (10 μmol/L) or ETYA (30 μmol/L) was added 20 minutes prior to the addition of AA. Incubations were stopped by the addition of ethanol to a final concentration of 15%. The buffer was collected and extracted on C18 solid‐phase extraction columns as described previously.^27^ Metabolites were evaporated under nitrogen and stored at −40°C until analysis by high‐performance liquid chromatography (HPLC).

### Reverse‐Phase HPLC

AA metabolites were resolved by reverse‐phase HPLC (Nucleosil‐C18 column, 5 μm, 4.6×250 mm).^27^ The program consisted of a 40‐minute linear gradient from 50% solvent B (acetonitrile containing 0.1% glacial acetic acid) in solvent A (water) to 100% solvent B. The flow rate was 1 mL/min. Column eluates were collected in 0.2‐mL fractions. Absorbance was monitored at 205 or 235 nm, and radioactivity of each fraction was determined by liquid scintillation spectrometry.

### Liquid Chromatography‐Electrospray Ionization Mass Spectrometry

HCAECs were plated onto 10‐cm petri dishes and grown to 70% to 80% confluence. Cells were incubated with 10 μmol/L AA for 10 minutes and then with 10 μmol/L A‐23187 for another 15 minutes at 37°C. The buffer was collected, and internal standards were added, followed by extraction steps as described above. The chemical identity of the major metabolites was analyzed by liquid chromatography‐electrospray ionization mass spectrometry using a Waters 2695 liquid chromatograph and a Waters Micromass Quattro Micro API mass spectrometer (Waters Corporation) as previously described.^28^ Fractions were chromatographed using a Kromasil C‐18 (5 μm, 2×250 mm) column. The mass spectrometer was operated in negative ion mode.

### Patch‐Clamp Recording of TRPV4 Currents

Single‐channel currents were recorded with an Axopatch 200B or an EPC‐7 amplifier as previously described.^6^ The pipette solution contained (in mmol/L) 140 NaCl, 1 MgCl_2_, and 10 HEPES (pH 7.4 with CsOH). The bath solution for both cell‐attached and inside‐out patches was composed of (in mmol/L) 135 KCl, 0.8 CaCl_2_, 1 MgCl_2_, 5 glucose, 5 EGTA, and 5 HEPES. The pH of the bath solution was adjusted to 7.3 with CsOH (final Cs+ concentration of 4 mmol/L) and KOH. Channel currents were recorded for at least 3 to 6 minutes under control conditions and after treatment with 4α‐PDD (1 μmol/L) or AA (1 μmol/L). All chemicals were applied by bath perfusion at the rate of 1 mL/min. Single‐channel currents were sampled at 3 to 5 kHz and filtered at 1 kHz and analyzed using pClamp 10 software. Experiments were performed at room temperature.

### Chemicals

GSK1016790A was kindly provided by GlaxoSmithKline Pharmaceuticals. RN‐1734 was obtained from Menai Organics Ltd (UK). HC‐067047 and PKI were obtained from Tocris (Bristol, UK). U46619, ETYA, 17‐ODYA, MS‐PPOH, and all 4 isomers of EETs were obtained from Cayman (Ann Arbor, MI). All other chemicals, including AA (sodium salt, from porcine liver, purity ≥99% by capillary GC), 4α‐PDD, GF 109203X, TRAM‐34, apamin, and valinomycin were obtained from Sigma‐Aldrich (St. Louis, MO). For some calcium assay protocols, AA was further purified by HPLC prior to use, and results were found to be similar to those obtained from the original AA. The following fluorescence probes were used in this study: fura‐2 AM (Molecular Probes) and PMPI (Molecular Devices). PMPI is a proprietary component of the FLIPR Membrane Potential Assay Kit (R8128). Stock solutions of GSK1016790A, 4α‐PDD, RN‐1734, TRAM‐34, valinomycin, and GF 109203X were prepared in DMSO. AA, U46619, ETYA, 17‐ODYA, MS‐PPOH, and all 4 isomers of EETs were dissolved in double‐distilled ethanol. Other chemicals such as apamin and PKI were dissolved in distilled H_2_O. All concentrations represent final molar concentrations in the bath. The final concentration of DMSO and ethanol in the bath was <0.05% to 0.1%; vehicle control studies indicated that the final concentration of DMSO and ethanol had no effect on the vascular or cellular responses examined in this study.

### Data Analysis

Vessels from each patient were examined with vehicle (control) and then with a pharmacological inhibitor. This experimental design allowed for paired analysis, thereby minimizing potential effects of other factors such as underlying diseases, age, and sex. Statistical comparisons of the percent of vasodilation under different treatments were performed by 2‐way (factor) repeated‐measures ANOVA (factor 1, doses; factor 2, different treatments such as vehicle or an inhibitor), followed by the Holm‐Sidak test for pairwise comparisons or comparisons versus a control. When no interaction between dose and treatment was detected, the overall dose responses between control and inhibitor groups were tested; for nonparallel profiles with a significant interaction between dose and treatment, responses to vehicle and an inhibitor were compared at each individual dose. For all other nonvascular experiments, significant differences between mean values were evaluated by paired or unpaired *t* tests or 1‐way ANOVA followed by the Holm‐Sidak multiple comparison test, where appropriate. In addition, a nonparametric test (Wilcoxon–Mann–Whitney rank sum test) was used for some 2‐sample experiments with a small sample size. All procedures were performed using the statistical analysis programs provided in SigmaPlot, version 12. *P* values <0.05 weres considered statistically significant. Data are presented as mean±SEM. For all vessel function studies, n indicates the number of patients.

## Results

### Role of TRPV4 and K^+^ Channels in AA‐Induced Dilation of HCAs

**Figure 1.**
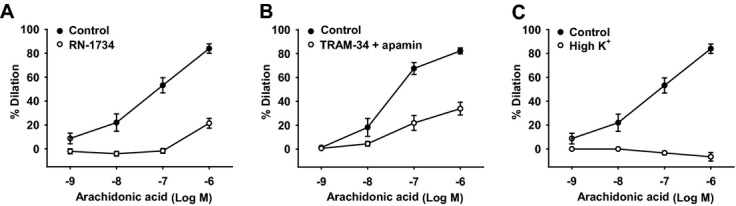
Role of TRPV4 and K^+^ channels in AA‐induced dilation of HCAs. A, AA dilated HCAs in a concentration‐dependent manner. The dilation was markedly inhibited by the TRPV4 antagonist RN‐1734 (20 μmol/L). B, AA‐induced dilation was inhibited by the combination of TRAM‐34 (1 μmol/L) and apamin (1 μmol/L), which selectively inhibit intermediate‐conductance K_Ca_ (IK_Ca_) and small‐conductance K_Ca_ (SK_Ca_) channels, respectively. C, AA‐induced dilation was eliminated by high extracellular K^+^ (80 mmol/L); n=5 to 7 patients/each group. *P<0.05 vs control. TRPV4 indicates transient receptor potential vanilloid 4; AA, arachidonic acid; HCA, human coronary artery.

We have shown previously that AA induces endothelium‐dependent dilation of HCAs through an EDH‐related mechanism; however, how AA elicits this EDH‐related dilation remains unclear.^9^ We first investigated the effect of TRPV4 blockade on vasodilator responses to AA using isolated coronary arterioles. In HCAs preconstricted with endothelin‐1, AA (10^−9^ to 10^−6^ mol/L) caused concentration‐dependent dilation (Figure 1A; dilation at 10^−6^ mol/L, 84±4%, n=7). The dilation was significantly attenuated by the TRPV4 antagonist RN‐1734 (20 μmol/L; 21±4%, n=5, *P*<0.05 versus control). To examine the specific Ca^2+^‐activated K^+^ (K_Ca_) channels involved in AA‐induced dilation, HCAs were incubated with TRAM‐34 (1 μmol/L) and apamin (1 μmol/L), which selectively inhibit IK_Ca_ and SK_Ca_ channels, respectively. As shown in Figure 1B, the dilation to AA was inhibited by the combination of TRAM‐34 and apamin (dilation at 10^−6^ mol/L, 34+5% versus 82±3% of control; n=5 each; *P*<0.05). Furthermore, AA‐induced dilation was eliminated by high K^+^ (80 mmol/L; −7±4%, n=5, *P*<0.05 versus control), confirming that this dilation involves an EDH‐dependent mechanism (Figure 1C). RN‐1734 did not affect baseline vascular tone, endothelin‐1‐induced preconstriction, or bradykinin‐induced dilation of HCAs (data not shown). Together, these results indicate that TRPV4 and IK_Ca_ and SK_Ca_ channels are involved in AA‐induced dilation of HCAs.

### AA‐Induced Increase of [Ca^2+^]_i_ in HCAECs

**Figure 2.**
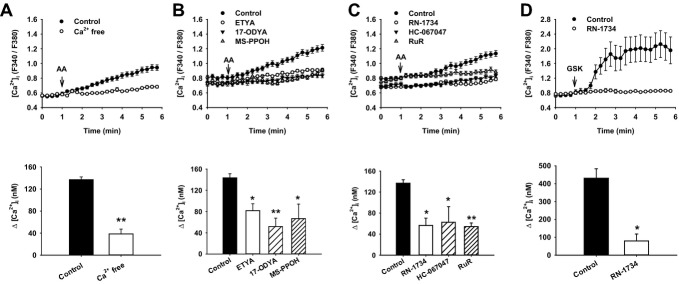
AA‐induced increase of [Ca^2+^]_i_ in native HCAECs. A, AA (3 μmol/L) increased [Ca^2+^]_i_, which was nearly eliminated in the absence of extracellular Ca^2+^ (Ca^2+^ free). B, AA‐elicited [Ca^2+^]_i_ increase was inhibited by 3 CYP pathway inhibitors: ETYA (30 μmol/L), 17‐ODYA (10 μmol/L), and MS‐PPOH (30 μmol/L). C, Treatment of cells with TRPV4 antagonists RN‐1734 (20 μmol/L), HC‐067047 (1 μmol/L), and ruthenium red (RuR; 1 μmol/L) also markedly inhibited AA‐induced [Ca^2+^]_i_ elevation. D, The TRPV4 agonist GSK1016790A (GSK; 10 nmol/L) increased [Ca^2+^]_i_, which was inhibited by RN‐1734. All data represent mean±SEM of ≥60 cells analyzed in 3 to 5 independent experiments. *P<0.05, **P<0.01 versus control. AA indicates arachidonic acid; ETYA, eicosatetraynoic acid; 17‐ODYA, 17‐octadecynoic acid; MS‐PPOH, N‐(methylsulfonyl)‐2‐(2‐propynyloxy)‐benzenehexanamide; HCAEC, human coronary artery endothelial cell; CYP, cytochrome P450; TRPV4, transient receptor potential vanilloid 4.

To examine whether AA activates endothelial TRPV4 channels, we measured [Ca^2+^]_i_ in HCAECs using a fura‐2 Ca^2+^ assay. As shown in Figure 2A (upper panel), AA (3 μmol/L) induced a gradual increase in [Ca^2+^]_i_, as indicated by a time‐dependent increase of the fluorescence ratio of F_340_ versus F_380_ (F_340_/F_380_) from 0.71±0.03 to 1.07±0.06. The average increase in [Ca^2+^]_i_ after 5 minutes’ exposure of AA was 137±5 nmol/L (Figure 2A, bottom panel). AA‐induced elevation of [Ca^2+^]_i_ was nearly eliminated in the absence of extracellular Ca^2+^ (39±9 nmol/L, *P*<0.01 versus control; Figure 2A), indicating that the increase of [Ca^2+^]_i_ is dependent on Ca^2+^ influx from the extracellular space but not on Ca^2+^ release from intracellular Ca^2+^ stores.

Next, we examined whether the AA‐elicited Ca^2+^ response is mediated through AA itself or CYP epoxygenase metabolites of AA. Preincubation of cells with either of 3 CYP inhibitors—ETYA (30 μmol/L), 17‐ODYA (10 μmol/L), or MS‐PPOH (30 μmol/L)—for 20 minutes markedly inhibited AA‐induced [Ca^2+^]_i_ elevation (Figure 2B). The Ca^2+^ influx induced by AA was also greatly attenuated by each of the 3 commercially available TRPV4 channel antagonists, RN‐1734 (20 μmol/L), HC‐067047 (1 μmol/L), and ruthenium red (1 μmol/L) (Figure 2C). To confirm the functional expression of TRPV4 in HCAECs, we tested the effect of the TRPV4 agonist GSK1016790A on [Ca^2+^]_i_. As shown in Figure 2D, GSK1016790A (10 nmol/L) robustly increased [Ca^2+^]_i_, with a maximal [Ca^2+^]_i_ increase of 430±53 nmol/L. In the presence of RN‐1734 (20 μmol/L), the [Ca^2+^]_i_, increase in response to GSK was markedly reduced (79±22 nmol/L, *P*<0.05 versus control). Collectively, these results suggest that AA activates TRPV4 to increase endothelial [Ca^2+^]_i_ and that AA‐induced TRPV4 activation may involve CYP‐catalyzed AA metabolism.

### Ca^2+^ Responses in hTRPV4‐Overexpressing HCAECs

**Figure 3.**
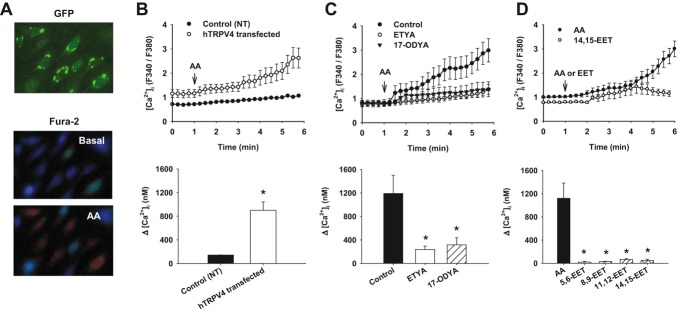
Ca^2+^ responses in hTRPV4‐overexpressing HCAECs. A, Representative images of endothelial cells expressing hTRPV4‐GFP (top) and corresponding cells loaded with fura‐2 before (middle) and after (bottom) AA treatment. B, Compared with nontransduced (NT) cells, AA (3 μmol/L) elicited augmented [Ca^2+^]_i_ elevation in hTRPV4‐expressing cells. C, The AA‐induced Ca^2+^ response was inhibited by CYP pathway inhibitors ETYA (30 μmol/L) and 17‐ODYA (10 μmol/L). D, None of 4 isomeric EETs (3 or 10 μmol/L in some experiments) showed substantial activation of TRPV4 channels compared with AA. All data represent mean±SEM of ≥60 cells analyzed in 3 to 5 independent experiments. *P<0.05 versus control. hTRPV4 indicates human transient receptor potential vanilloid 4; HCAEC, human coronary artery endothelial cell; GFP, green fluorescent protein; AA, arachidonic acid; CYP, cytochrome P450; EET, epoxyeicosatrienoic acid.

To further test the contribution of TRPV4 in the AA‐induced [Ca^2+^]_i_ increase and the role of CYP metabolites as endogenous mediators of TRPV4 activation, we examined Ca^2+^ responses in HCAECs overexpressing hTRPV4. Using a lentiviral vector‐mediated transduction system, we obtained a transduction efficiency of close to 90% to 100%, as indicated by the representative images of GFP‐positive and fura‐2‐loaded endothelial cells (Figure 3A). Compared with control nontransduced HCAECs, the AA (3 μmol/L)‐induced increase of [Ca^2+^]_i_ was markedly enhanced in hTRPV4‐overexpressing endothelial cells (899±142 versus 143±3 nmol/L of control, *P*<0.05; Figure 3B). The effect of AA on TRPV4‐mediated Ca^2+^ entry was concentration dependent, and the minimal effective concentration of AA was close to 1 μmol/L (data not shown). In contrast, the fatty acid analogues palmitate (0.3 to 3 μmol/L) and arachidic acid (0.1 to 10 μmol/L) did not increase [Ca^2+^]_i_ in hTRPV4‐expressing HCAECs, indicating that AA‐induced TRPV4 activation is not a result of nonspecific fatty acid effects (Figure S1). Similar to nontransduced HCAECs, both ETYA (30 μmol/L) and 17‐ODYA (10 μmol/L) inhibited the AA‐elicited increase in [Ca^2+^]_i_ in hTRPV4‐overexpressing HCAECs. Again, these results are consistent with previous reports that AA activates TRPV4 through CYP epoxygenase metabolites.^19–22^ CYP inhibitors such as 17‐ODYA did not affect the TRPV4 agonist 4α‐PDD (1 μmol/L)‐induced Ca^2+^ response (Δ[Ca^2+^]_i_, 2507±487 versus 2287±303 nmol/L of controls; n=5; *P*>0.05) in hTRPV4‐expressing HCAECs; therefore, it is unlikely that these CYP inhibitors nonspecifically affect TRPV4 function. We next examined the direct effect of the CYP epoxygenase metabolites of AA, EETs, on [Ca^2+^]_i_ in hTRPV4‐overexpressing HCAECs. Surprisingly, compared with AA, none of the 4 isomers of EETs (3 or 10 μmol/L in some experiments) showed substantial activation of TRPV4 at concentrations similar to or higher than that of AA (Figure 3D). Moreover, the specific EET antagonist 14,15‐EEZE (10 μmol/L) did not inhibit the AA‐induced [Ca^2+^]_i_ increase (Figure S2).

### CYP Metabolism of AA in HCAECs

**Figure 4.**
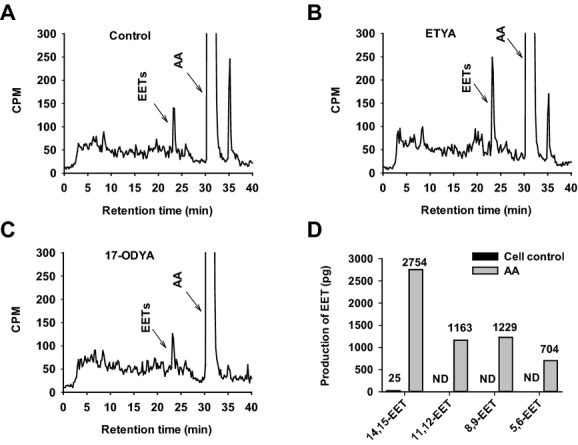
Cytochrome P450 metabolism of AA in HCAECs. A, HCAECs were incubated with [^14^C]AA, metabolites of [^14^C]AA were extracted and resolved by reverse‐phase HPLC, and radioactivity of each fraction was determined by liquid scintillation spectrometry. Migration times of known standards are indicated. A major peak eluting at 22 to 24 minutes comigrated with the EET standard. This EET peak was not inhibited by CYP inhibitors ETYA (B; 30 μmol/L) or 17‐ODYA (C; 10 μmol/L). D, LC‐mass spectrometric analysis confirmed the production of 4 isomers of EETs— 14,15‐, 8,9‐, 11,12‐, and 5,6‐EET—in cells treated with AA (10 μmol/L). Endogenous EETs in nontreated cells were at low levels or not detectable (ND). CPM indicates counts per minute; AA, arachidonic acid; HCAEC, human coronary artery endothelial cell; HPLC, high‐performance liquid chromatography; EET, epoxyeicosatrienoic acid; CYP, cytochrome P450.

To examine whether coronary ECs metabolize AA to EETs, HCAECs were incubated with [^14^C]AA with and without the CYP450 inhibitors ETYA (30 μmol/L) or 17‐ODYA (10 μmol/L). Metabolites of AA were then resolved using reverse‐phase HPLC, and retention times of radioactive metabolites were compared with known standards. As shown in Figure 4A, a major peak eluting at 22 to 24 minutes comigrated with the EET standard. LC‐mass spectrometric analysis confirmed that 1 or more isomers of EETs were produced in HCAECs treated with AA (Figure 4D). However, the EET peak was not significantly inhibited by ETYA (Figure 4B) or 17‐ODYA (Figure 4C), with averaged values of radioactivity (counts per minute) of 164±57 (n=2) and 113±12 (n=2), respectively, versus 150±29 (n=5) of the control. These findings, together with the results obtained with the Ca^2+^ assay, suggest that the release of CYP450 epoxygenase metabolites EETs may not be the main mechanism responsible for AA‐induced and TRPV4‐mediated Ca^2+^ responses in HCAECs.

### Modulation of TRPV4‐Mediated Ca^2+^ Influx by Membrane Potential

To further explore how AA activates endothelial TRPV4 channels, we examined 3 potential mechanisms that may contribute to TRPV4 activation: regulation of cell membrane potential, channel protein phosphorylation, and direct gating of channel activity.

**Figure 5.**
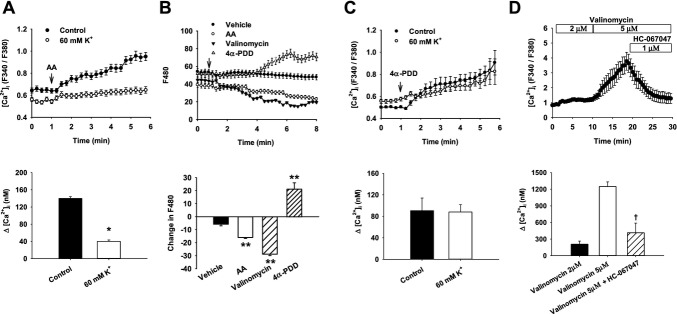
Modulation of TRPV4‐mediated Ca^2+^ influx by membrane potential. A, AA (3 μmol/L)‐elicited [Ca^2+^]_i_ increase was blunted in the presence of 60 mmol/L extracellular K^+^. B, AA induced membrane hyperpolarization in HCAECs, as indicated by a decrease in PMPI fluorescence intensity (F480). In contrast, the TRPV4 agonist 4α‐PDD (3 μmol/L) depolarized the membrane potential. Valinomycin (K^+^‐selective ionophore, 2 μmol/L) was used as a positive control to indicate membrane hyperpolarization. C, The TRPV4 specific agonist 4α‐PDD (5 μmol/L) increased [Ca^2+^]_i_ in HCAECs, and the increase was not affected by 60 mmol/L K^+^. D, Membrane hyperpolarization by valinomycin resulted in Ca^2+^ influx in hTRPV4‐expressing HCAECs, which was inhibited by HC‐067047. The data represent mean±SEM of ≥60 cells analyzed in 3 to 5 independent experiments. *P<0.05 vs control, **P<0.01 vs vehicle, ^†^*P*<0.05 vs valinomycin (5 μmol/L). TRPV4 indicates transient receptor potential vanilloid 4; AA, arachidonic acid; 4α‐PDD, 4α‐phorbol‐12,13‐didecanoate; HCAEC, human coronary artery endothelial cell; PMPI, plasma membrane potential indicator.

As previously described in Figure 1, AA‐induced dilation of HCAs was abolished by high K^+^ and was markedly inhibited by TRAM‐34 plus apamin, indicating that AA‐induced dilation requires membrane hyperpolarization. It is possible that AA‐elicited TRPV4 activation is dependent on membrane hyperpolarization or that AA activates TRPV4, which in turn induces membrane hyperpolarization through K_Ca_ channel activation. To test these possibilities, we measured the effect of high K^+^ on the AA‐induced [Ca^2+^]_i_ increase in HCAECs. As shown in Figure 5A, AA‐induced [Ca^2+^]_i_ was largely inhibited by a high K^+^ solution. Similarly, the effect of AA on [Ca^2+^]_i_ was inhibited by depolarization induced by the cation ionophore gramicidin (data not shown). Membrane depolarization had no effect on [Ca^2+^]_i_ under resting conditions. These results suggest that membrane potential regulates AA‐elicited and TRPV4‐mediated Ca^2+^ entry. Using the plasma membrane potential indicator PMPI, we found that AA induced membrane hyperpolarization, as indicated by a decrease in fluorescence intensity at 480 nm (Figure 5B; *P*<0.05 versus vehicle). The TRPV4 agonist 4α‐PDD (3 μmol/L) induced membrane depolarization (Figure 5B; *P*<0.05 versus vehicle), an effect consistent with the opening of a cation‐permeable channel like TRPV4. In contrast to its effect on AA, high K^+^ did not affect the Ca^2+^ response to the synthetic TRPV4 agonist 4α‐PDD (Figure 5C). However, in hTRPV4‐expressing HCAECs, membrane hyperpolarization by valinomycin (2 to 5 μmol/L; K^+^‐selective ionophore) induced an increase in [Ca^2+^]_i_ that was subsequently inhibited by the TRPV4 antagonist HC‐067047 (*P*<0.05 versus valinomycin; Figure 5D), indicating that the membrane potential modulation of TRPV4‐mediated Ca^2+^ entry may be stimulus specific. Collectively, these data indicate that AA induces endothelial K^+^ activation and membrane hyperpolarization and that this membrane hyperpolarization is essential for TRPV4‐mediated Ca^2+^ entry in response to AA stimulation.

### Regulation of TRPV4 Activation by Protein Phosphorylation

**Figure 6.**
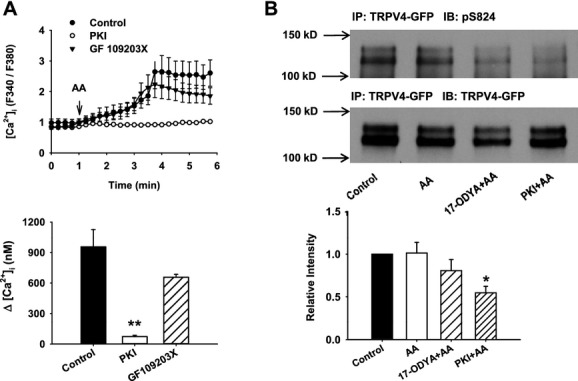
Regulation of TRPV4 activation by protein phosphorylation. A, In hTRPV4‐expressing HCAECs, the AA‐induced [Ca^2+^]_i_ increase was almost abolished by the PKA inhibitor PKI (1 μmol/L), whereas this AA response was only partially but not significantly attenuated by the PKC inhibitor GF 109203X (1 μmol/L). B, TRPV4 is phosphorylated at serine‐824 in unstimulated HCAECs expressing the hTRPV4‐GFP fusion protein. The level of serine‐824 phosphorylation was not further increased by AA (3 μmol/L). PKI (1 μmol/L) significantly inhibited serine‐824 phosphorylation. Endothelial cells were treated with the indicated reagents, and TRPV4‐GFP was immunoprecipitated with GFP antibodies and detected with pS824 antibodies (upper panel). The same membrane was reprobed with GFP antibodies to detect total TRPV4 protein (middle panel). All data represent mean±SEM from 3 to 5 independent experiments. At least 60 cells analyzed in (A). *P<0.05, **P<0.01 vs control. TRPV4 indicates transient receptor potential vanilloid 4; HCAEC, human coronary artery endothelial cell; AA, arachidonic acid; PKA, protein kinase A; PKI, protein kinase A inhibitor; IP, immunoprecipitation; IB, immunoblotting; GFP, green fluorescent protein.

In hTRPV4‐overexpressing HCAECs, the PKA inhibitor PKI (1 μmol/L) markedly inhibited the AA‐induced [Ca^2+^]_i_ increase (74±13 versus 955±170 nmol/L of control, *P*<0.01; Figure 6A), whereas the PKC inhibitor GF109203X (1 μmol/L) slightly attenuated, but not significantly, the AA response (658±29 nmol/L, *P*>0.05). The PKA activator forskolin (10 μmol/L) did not increase endothelial [Ca^2+^]_i_ (173±16 versus 202±23 nmol/L of baseline, n=7). These results indicate that protein phosphorylation is required for the activation of TRPV4 channels by AA but may not directly open TRPV4 channels. To directly examine whether TRPV4 channels are phosphorylated, protein lysates of hTRPV4‐expressing HCAECs were immunoprecipitated with an antibody against the GFP peptide tag and then detected with an anti‐pSer824 antibody. As shown in Figure 6B, TRPV4 channels in HCAECs displayed a high level of serine phosphorylation under basal conditions. Treatment of cells with AA (3 μmol/L) did not induce significant additional phosphorylation of TRPV4. PKI (1 μmol/L) inhibited basal phosphorylation of TRPV4 at serine‐824 (relative intensity, 0.55±0.07, *P*<0.05 versus control), indicating that PKA is mainly responsible for TRPV4 phosphorylation in coronary ECs. This phosphorylation was slightly inhibited, although not statistically significantly, by 17‐ODYA (10 μmol/L; 0.80±0.13); therefore, we cannot exclude the possibility that endogenous AA (and/or its metabolites) induces PKA‐mediated protein phosphorylation of TRPV4 channels.

### Effect of AA on TRPV4 Single‐Channel Currents in HCAECs

**Figure 7.**
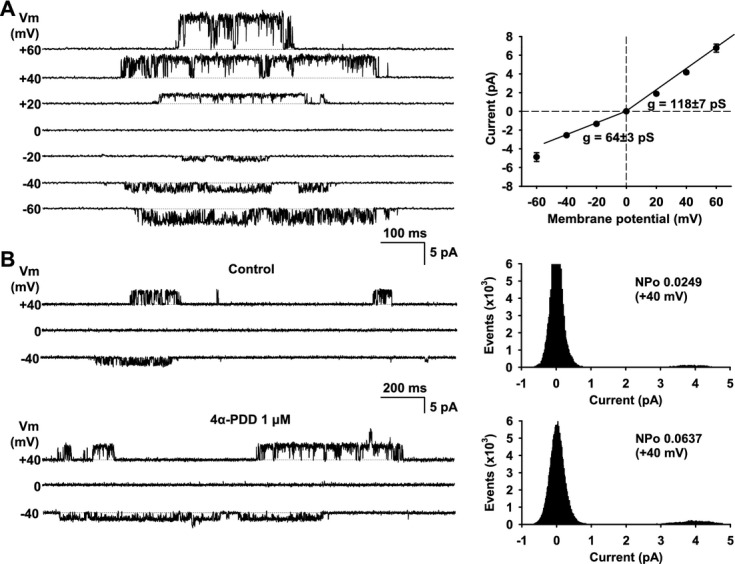
Single‐channel properties of hTRPV4 overexpressed in HCAECs. A, TRPV4 single‐channel currents (left) were recorded from a cell‐attached patch at the indicated membrane potentials (Vm). Right panel depicts average current–voltage relationship for single‐channel currents, with a calculated slope conductance determined over voltage ranges of 0 to 60 and −40 to 0 mV, respectively. Data are mean±SE from 5 membrane patches. B, Single‐channel activity of TRPV4 (left) was low under basal conditions but markedly increased following bath perfusion of the TRPV4 agonist 4α‐PDD (1 μmol/L). Shown on the right are the corresponding amplitude histograms in relation to open‐state probability (NPo). Data are representative of >5 membrane patches. Dashed lines indicate current level when the channel is closed. Cell‐attached recordings were obtained with a normal extracellular solution (140 Na^+^, 5 Cs^+^) in the pipette and a high‐K^+^ (140 K^+^) bath solution to zero the cell membrane potential. TRPV4 indicates transient receptor potential vanilloid 4; HCAEC, human coronary artery endothelial cell.

To directly examine the effect of AA on TRPV4 activity, we measured TRPV4 single‐channel currents using the patch‐clamping technique. HCAECs overexpressing hTRPV4 were used in these studies in an attempt to record TRPV4 single‐channel currents at a much higher success rate in those cells (>70%) than in native HCAECs (<5% to 10%). As illustrated in Figure 7A, unitary single‐channel currents were recorded from a cell‐attached patch on hTRPV4‐overexpressing HCAECs. With a normal extracellular solution (140 Na^+^, 5 Cs^+^) in the pipette and a high‐K^+^ (140 K^+^) bath solution to null the cell membrane potential, single‐channel current openings were detected at different patch potentials with a reverse potential of 0 mV. The calculated slope conductances in the membrane potential ranges of 0 to 60 and −40 to 0 mV averaged 118±7 (n=5) and 64±3 (n=5) pS, respectively. The channel open‐state probability (NPo) was usually low under control nonstimulated conditions (Figure 7B). NPo was markedly increased with a variable delay (2 to 5 minutes) following bath perfusion of the TRPV4‐selective agonist 4α‐PDD (1 μmol/L). Channel activity was not significantly altered when the cell was held at different membrane potentials, and 4α‐PDD activated similar single‐channel currents in inside‐out patches of ECs (data not shown). These results indicate that the single‐channel currents recording from hTRPV4‐expressing HCAECs display electrophysiological and pharmacological properties consistent with those of TRPV4 single‐channel currents reported in HEK‐293 and other cell lines.^29^

**Figure 8.**
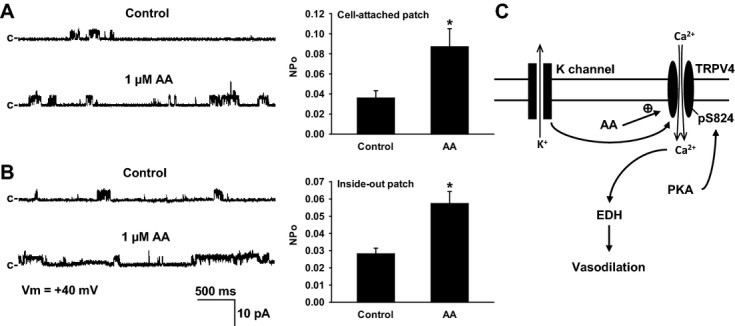
Effect of AA on hTRPV4 single‐channel currents in HCAECs. AA (1 μmol/L) increased TRPV4 single‐channel currents in both cell‐attached patches (A) and inside‐out patches, which lacked intracellular constituents (B). Left, representative recordings at the indicated membrane potential (Vm). Note that c is the current level when the channel is closed. Right, summary of channel open‐state probability (NPo). Cell‐attached and inside‐out patch recordings before and after treatment with AA were performed with a normal extracellular solution (140 Na^+^,5 Cs^+^) in the pipette and a high‐K^+^ (140 K^+^) solution in the bath. n=6 patches/each group. *P<0.05 vs control. C, Possible mechanism for AA‐induced Ca^2+^ entry through TRPV4 channels in human coronary endothelial cells and subsequent dilation of coronary arterioles. AA indicates arachidonic acid; TRPV4, transient receptor potential vanilloid 4; HCAEC, human coronary artery endothelial cell; PKA, protein kinase A; EDH, endothelium‐dependent hyperpolarization.

In cell‐attached patches of hTRPV4‐expressing HCAECs, the addition of 1 μmol/L AA to the bath solution increased the activity of TRPV4 single‐channel currents (NPo, 0.036±0.007 before AA versus 0.087±0.018 after AA; n=6 each; *P*<0.05; Figure 8A). The activation of TRPV4 channels by AA showed little desensitization during 10 to 15 minutes of stimulation (data not shown). In inside‐out patches of hTRPV4‐expressing endothelial cells (Figure 8B), AA (1 μmol/L) also increased the TRPV4 channel opening (NPo, 0.028±0.003 before AA versus 0.057±0.007 after AA; n=6 each; *P*<0.05). These results provide strong evidence that AA can directly activate TRPV4 channels in HCAECs, an effect that does not seem to require intermediate signaling or conversion of AA into active metabolites.

## Discussion

This study is the first to investigate the role of endothelial TRPV4 channels in the vasodilator response to AA, a potentially important mediator of EDH‐related vasodilation in human coronary microcirculation. The major novel findings of the present study are 2‐fold (Figure 8C). First, AA‐induced EDH‐related dilation of HCAs requires endothelial TRPV4 channels. Second, AA induces TRPV4‐mediated Ca^2+^ entry in coronary ECs, and the initiation of this Ca^2+^ response involves several coordinated intracellular events, including membrane hyperpolarization, PKA‐mediated basal phosphorylation of TRPV4, and a direct effect of AA on TRPV4 channel activity. These results, together with our previous findings that TRPV4 channels are predominantly expressed in the endothelium of HCAs and that opening of TRPV4 induces potent endothelium‐dependent vasodilation,^18^ suggest that the TRPV4 channel in ECs serves as a previously unidentified signaling component in mediating AA‐induced EDH‐dependent vasodilation. In addition, the finding that TRPV4‐mediated Ca^2+^ entry in ECs is regulated by several mechanisms provides new insight into the complex polymodal regulation of this channel, a characteristic that may be crucial for TRPV4‐mediated responses to physiological stimuli such as shear stress or receptor agonists.

### Endothelial TRPV4 in AA‐Induced EDH‐Dependent Dilation

EETs, the CYP epoxygenase metabolites of AA, have been proposed to function as EDHFs mediating non‐NO‐ and non‐PGI_2_‐mediated vasodilation in arteries from various species and vascular beds, although their mechanisms of action remain incompletely understood and may vary in different preparations.^2^ Traditionally, EETs are thought to be released from the endothelium and act as transferable factors that hyperpolarize and relax smooth muscle cells (SMCs), as has been demonstrated in arteries such as the bovine coronary artery.^2^ In contrast to this “transferrable factor” mechanism, other studies have indicated that in arteries such as the rat hepatic artery, EETs act as autocrine factors in ECs to increase [Ca^2+^]_I_, which consequently leads to activation of the K_Ca_ channels expressed in ECs, specifically SK_Ca_ and IK_Ca_ channels. The opening of these endothelial K_Ca_ channels results in hyperpolarization of the ECs, which in turn, through a “transfer of hyperpolarization” or EDH mechanism, causes hyperpolarization and relaxation of SMCs.^2^ We previously used HCAs to show that AA produces potent endothelium‐dependent vasodilation that is largely inhibited by nonselective K_Ca_ blockers, indicating AA‐induced EDH‐dependent dilation in this vascular bed.^9^ In the present study, we have extended these observations by demonstrating that AA‐induced dilation involves the activity of endothelial IK_Ca_ and SK_Ca_ channels. Furthermore, we have provided the first evidence that activation of endothelial TRPV4 channels plays a key role in AA‐induced EDH‐related dilation in HCAs.

The precise mechanisms by which TRPV4 channels interact with IK_Ca_ and SK_Ca_, and other potential signaling components to elicit the EDH component of dilation in HCAs remain to be determined. Although it is possible that TRPV4‐mediated Ca^2+^ entry activates IK_Ca_ or SK_Ca_ channels, which could subsequently elicit an EDH mechanism of dilation, TRPV4‐mediated Ca^2+^ entry may also induce the release of some transferable factors, such as H_2_O_2_ or metabolites of AA other than EETs (see below), to induce relaxation of SMCs. Given the robust expression of TRPV4 and other TRP channels in endothelial cells of various species,^17^ activation of these Ca^2+^‐permeable channels may act as a conserved mechanism mediating EDH‐dependent vasodilation. Indeed, AA‐induced dilation was also reduced in mesenteric small arteries from TRPV4‐knockout mice compared with wild‐type controls (authors’ unpublished observations).

### Mechanisms of AA‐Induced TRPV4 Activation in Coronary ECs

Previous studies have indicated that AA and/or its CYP epoxygenase metabolites, EETs, activate TRPV4 channels in murine vascular cells and in cell lines heterologously expressing murine TRPV4 channels; however, the metabolites (in particular, the specific isomers of EETs) responsible for TRPV4 activation remain controversial.^19–22^ In the present study, we performed an in‐depth analysis of the effect and mechanism of action of AA on TRPV4 activity using HCAECs. We found that AA increased [Ca^2+^]_i_ in HCAECs and this increase was inhibited by different TRPV4 antagonists. The CYP inhibitors (17‐ODYA and ETYA) also largely blocked the Ca^2+^ response induced by AA in both native and TRPV4‐transfected HCAECs. Although these data are consistent with the view that AA activates TRPV4 through its CYP metabolites (presumably EETs), results from subsequent studies suggest that an alternative interpretation is needed. First, CYP inhibitors did not affect the production of EETs in coronary ECs, indicating that inhibition of the AA‐induced Ca^2+^ response may not be related to reduced EET synthesis as expected for these blockers. Because CYP inhibitors such as 17‐ODYA did not affect the Ca^2+^ response to the TRPV4 agonist 4α‐PDD, it is unlikely that these CYP inhibitors nonspecifically affect TRPV4 function. Instead, these inhibitors may antagonize the action of AA at the TRPV4 protein level. Second, blockade of EET action by the EET antagonist 14,15‐EEZE did not affect AA‐induced TRPV4‐dependent Ca^2+^ entry. Third, none of the 4 isomers of EETs were able to activate TRPV4 to the same extent as that activated by AA. Although EETs at micromolar concentrations did induce a small [Ca^2+^]_i_ increase in hTRPV4‐overexpressing HCAECs, it is difficult to envisage that coronary ECs can efficiently produce EETs from exogenously applied AA (1 to 3 μM) up to those or higher concentrations to elicit significant TRPV4 activation. Finally, our patch‐clamp studies demonstrated that AA activated TRPV4 channels in cell‐attached as well as inside‐out patches of coronary ECs. Therefore, we propose that in human coronary ECs, EETs may not be the main mediators of TRPV4 activation but instead AA itself may be responsible for TRPV4 activation, although EETs may play some modulatory roles in TRPV4 activation or TRPV4‐mediated Ca^2+^ signaling. It is of note that a putative arachidonate recognition sequence, which was shown to be important for AA‐induced activation of the TRP channel TRPM2 and the 2‐pore‐domain K^+^ channel TREK‐1, is present at a cytoplasmic domain of TRPV4 (amino acids 402 to 408 of hTRPV4).^29^

The reasons for the involvement of different mediators in AA‐induced TRPV4 activation in HCAs versus murine arteries^19–22^ remain to be determined but could be due to differences in species or vascular beds. In addition, further studies are required to understand the lack of effect of CYP inhibitors on EET synthesis in human coronary ECs. Similar to the results of this study, a previous report has also shown that 17‐ODYA and miconazole did not affect EET and DHET (dihydroxy eicosatrienoic acid) levels in rat tumor tissues.^30^ It is possible that EETs are produced from an unidentified CYP epoxygenase isoform (not sensitive to the CYP inhibitors used in this study) that is upregulated in coronary ECs, since the 2 known EET‐producing CYP epoxygenases (CYP2C9 and CYP2J2) are inhibited by H_2_O_2_,^10^ the key vasodilator factor in HCAs from patients with CAD. Alternatively, EETs could be generated from endogenous EET pools as esters of glycerophospholipids,^31^ and therefore the production of EETs will not be affected by CYP inhibitors. However, this later possibility seems unlikely since ^14^C‐labeled AA was used in the study of AA metabolism and all EETs measured by HPLC should be derived from exogenous ^14^C‐AA rather than from endogenous EET pools.

AA induces noncapacitative Ca^2+^ entry in other mammalian cells including vascular endothelial cells.^32^ In bovine aortic ECs, AA (at low dose <5 μmol/L) through a direct effect induced Ca^2+^ influx and activated several types of Ca^2+^‐permeable nonselective cationic (≤20 pS) currents.^33^ In HCAECs treated with TRPV4 antagonists, there was a small but consistent residual Ca^2+^ response to AA, indicating that AA may activate other Ca^2+^ entry channels. Future studies will determine the molecular identity of these AA‐activated Ca^2+^ channels.

Although there is evidence that TRPV4 or other TRP‐mediated Ca^2+^ entry leads to endothelial membrane hyperpolarization as a result of Ca^2+^‐induced activation of IK_Ca_ and/or SK_Ca_ channels,^22,34^ it remains unknown whether TRPV4‐mediated Ca^2+^ entry is conversely regulated by membrane hyperpolarization. In the present study, we found that AA‐induced TRPV4‐mediated Ca^2+^ influx in HCAECs was inhibited by membrane depolarization (induced by high K^+^) but was enhanced by membrane hyperpolarization (induced by valinomycin). However, membrane potentials did not significantly modulate single‐channel opening of TRPV4 channels, indicating that membrane hyperpolarization facilitates TRPV4‐mediated Ca^2+^ entry without affecting its open probability. Collectively, these results indicate that, in addition to its direct effect on TRPV4 activity, AA induces endothelial hyperpolarization and that this hyperpolarization is required for TRPV4‐mediated Ca^2+^ entry. These findings are consistent with those of other studies showing that membrane hyperpolarization increases Ca^2+^ influx in ECs, whereas depolarization has the opposite effect.^35–37^ However, not all studies support this membrane potential‐dependent regulation of endothelial Ca^2+^ entry.^38–40^ Therefore, the effect of membrane potential on endothelial Ca^2+^ entry may depend on the specific Ca^2+^ entry channel involved, and further investigation is need to elucidate the underlying mechanisms for this differential regulation.

A number of studies have shown that protein kinases such as PKA and PKC enhance the gating and phosphorylation of the TRPV4 channel,^16,26,41–42^ similarly to the phosphorylation‐induced sensitization of its close relative, TRPV1.^43^ For example, PKA phosphorylates the Ser‐824 residue of TRPV4 channels expressed in HEK‐293 and other cell lines, and this phosphorylation enhances the TRPV4‐mediated Ca^2+^ response to hypotonic cell swelling.^26,42^ Consistent with these previous studies, we found that the PKA inhibitor PKI almost abolished AA‐induced TRPV4‐mediated Ca^2+^ influx in coronary ECs. In addition, PKI markedly inhibited Ser‐824 phosphorylation of TRPV4 channels. Therefore, these data indicate that PKA‐mediated channel phosphorylation is an important regulator of TRPV4 activation by AA in coronary ECs, probably by sensitizing the channel to AA. In coronary ECs, TRPV4 channels exhibited substantial phosphorylation of Ser‐824 under basal conditions, and AA did not induce an apparent further increase in this phosphorylation. It remains to be determined whether AA enhances Ser‐824 phosphorylation of TRPV4 channels when coronary ECs are driven into a dephosphorylated state using pharmacological or molecular approaches. Nevertheless, PKA‐mediated phosphorylation of TRPV4 channels by itself does not seem to exert significant effect on the activity of TRPV4 channels in coronary ECs because forskolin (a PKA activator) did not increase basal [Ca^2+^]_i_ in these cells. Previous studies have also found that forskolin, which enhanced TRPV4 phosphorylation at Ser‐824, had little effect on basal [Ca^2+^]_i_ in TRPV4‐transfected HEK‐293 cells with a relatively low level of Ser‐824 phosphorylation under resting conditions.^26,42^ Together, these data indicate that PKA‐mediated phosphorylation plays an important role in TRPV4 activation by AA. This phosphorylation is most likely a prerequisite for AA‐induced activation of TRPV4 channels, but it itself does not activate the channels, as has been reported for ARC (arachidonate‐regulated Ca^2+^) channels.^44^ This mode of channel regulation by phosphorylation seems different from that of some vascular K^+^ channels.^45^ For example, phosphorylation by PKA or protein kinase G (PKG) by itself can directly activate the large‐conductance Ca^2+^‐activated K^+^ (BK_Ca_) channel by increasing the open probability of this channel.^45^

### Study Limitations

Using the fura‐2 Ca^2+^ assay in cultured human coronary ECs, we demonstrated that AA activated TRPV4 channels at 1 to 3 μmol/L, a concentration range similar to or lower than those used in other studies examining AA‐activated ion channels.^46^ These concentrations are higher than those used for cannulated vessel experiments in which the EC_50_ (the concentration of drug that produces a 50% maximal response) for AA‐induced vasodilation is in the nanomolar range. The discrepancy could be a result of differences in experimental conditions such as tissue culture or temperature that may have potentially affected the sensitivity of TRPV4 channels. Alternatively, AA at lower, submicromolar concentrations may induce smaller or localized Ca^2+^ increases in human coronary ECs, which may initiate intracellular signaling cascades leading to vasodilation. These elevations may not be detected in our fura‐2‐based global Ca^2+^ assay. Nevertheless, the concentrations of AA used in the cultured coronary ECs of the present study seem within the physiological range of AA endogenously produced in cells.^47–48^ For example, the release of 1% AA from esterified lipid pools in human platelets could give up to 50 μmol/L local concentrations before its release from the cells, and the percentage of AA released from stores in activated platelets is around 10%.^48^

Although EETs do not appear to be the main activator of TRPV4 channels in coronary ECs, involvement of other potential TRPV4‐activating metabolites derived from AA metabolism that are inhibited by CYP inhibitors (ETYA and 17‐ODYA) cannot be excluded and therefore will be pursued in future studies.

In coronary ECs, AA induced membrane hyperpolarization, and this hyperpolarization seems not to be related to the opening of TRPV4. The molecular mechanisms of this endothelial hyperpolarization elicited by AA remain to be explored. In addition, it remains to be determined whether potential hyperpolarizing channels are present in the endothelium of freshly isolated HCAs and whether they contribute to EDH‐related dilation in response to AA.

### Clinical Implications

Flow‐induced dilation and, to a lesser extent, agonist‐induced dilation of coronary arterioles from patients with CAD are mediated by a unique EDH‐dependent mechanism involving the release of H_2_O_2_ from ECs and subsequent smooth muscle hyperpolarization, whereas in the absence of CAD or its risk factors, other traditional endothelial relaxing factors (ie, NO and PGI_2_) play a more prominent role in dilation of coronary arterioles.^4^ The molecular mechanisms responsible for the switch from NO/PGI_2_‐ to H_2_O_2_‐mediated dilation in disease remain unknown. Our recent studies indicate that TRPV4‐mediated Ca^2+^ entry serves as an important signaling event leading to flow‐induced ROS release and vasodilation in HCAs.^18^ In the present study, we have provided evidence that AA, by regulating multiple intracellular pathways (without involving the CYP metabolites EETs), potently activates TRPV4 channels in coronary ECs. Given that a common early signaling event in ECs in response to agonist stimulation is activation of phospholipases and subsequent release of AA,^1–2^ the AA‐TRPV4‐H_2_O_2_ pathway may turn out to be an important mechanism in the regulation of coronary vascular tone in disease. This novel signal pathway may originate as an adaptive mechanism when other endothelial factors such as NO and PGI_2_ as well as EETs are impaired or lost in disease; it may also contribute to the progression of disease by recruiting H_2_O_2_, a ROS that has atherogenic properties and has been implicated in other cardiovascular diseases.
